# Knowledge, attitude, and practice of nephrologists on the decision for renal replacement therapy

**DOI:** 10.1186/s12889-023-15530-0

**Published:** 2023-04-05

**Authors:** Xiaofang Hu, Ming Yang, Xiangyi Li, Yu Chen, Shaxi Ouyang, Lin Li

**Affiliations:** 1 Department of Clinical Medicine, School of Medicine, Hunan Normal University, No. 371 Tongzipo Road, Yue-Lu District, Changsha, 410013 Hunan China; 2 Department of Nephrology, Zhuzhou Central Hospital, Zhuzhou, 412007 Hunan China; 3 Department of Nephrology, Hunan Provincial People’s Hospital, The First-Affiliated Hospital of Hunan Normal University, No. 61 Jie-Fang West Road, Fu-Rong District, Changsha, 410005 Hunan China

**Keywords:** Renal replacement therapy, Nephrologists, Knowledge, Attitude, Practice, Cross-sectional study

## Abstract

**Background:**

This study aimed to investigate the Knowledge, Attitude, and Practice (KAP) of nephrologists on the decision of renal replacement therapy (RRT), including peritoneal dialysis, hemodialysis, and kidney transplantation.

**Methods:**

This multicenter cross-sectional study was conducted on qualified nephrologists who volunteered to participate between July and August 2022 by using a self-administered questionnaire.

**Results:**

Among 327 nephrologists, the total knowledge, attitude, and practice scores were 12.03 ± 2.11/16, 58.39 ± 6.62/75, and 27.15 ± 2.74/30, respectively. Multivariate logistic regression analysis showed that the attitude score (peritoneal dialysis: OR = 1.19, 95%CI: 1.13–1.25, *P* < 0.001; hemodialysis: OR = 1.14, 95%CI: 1.09–1.19, *P* < 0.001; kidney transplantation: OR = 1.12, 95%CI: 1.07–1.16, *P* < 0.001), 41–50 years of age (peritoneal dialysis: OR = 0.45, 95%CI: 0.21–0,98, *P* = 0.045; hemodialysis: OR = 0.27, 95%CI: 0.12–0.60, *P* = 0.001; kidney transplantation: OR = 0.45, 95%CI:0.20–0.97, *P* = 0.042), and > 50 years of age (peritoneal dialysis: OR = 0.27, 95%CI: 0.08–0.84, *P* = 0.024; hemodialysis: OR = 0.45, 95%CI: 0.20–0.97, *P* = 0.042; kidney transplantation: OR = 0.24, 95%CI: 0.08–0.77, *P* = 0.016) were independently associated with the consideration score of peritoneal dialysis, hemodialysis, and kidney transplantation.

**Conclusion:**

Better attitudes may lead to more consideration by nephrologists when choosing between peritoneal dialysis, hemodialysis, and kidney transplantation and relatively less consideration by senior physicians when making decisions; in addition, having good knowledge and good attitudes may lead to better practice.

**Supplementary Information:**

The online version contains supplementary material available at 10.1186/s12889-023-15530-0.

## Background

Renal replacement therapy (RRT) is fluid removal and replacement for maintaining solute, acid–base, and electrolyte balance using dialysis and/or hemofiltration [1–3]. The types of venous RRT include continuous renal replacement therapy (CRRT), intermittent renal replacement therapy (IRRT), sustained low-efficiency dialysis (SLED), and peritoneal dialysis, which is more common in areas with limited resources and is easier than venous RRT to administer, but it does not allow for control of the fluid removal rate, and there are risks of protein loss, peritonitis, hyperglycemia, and potential respiratory impairment [1–3]. Some patients will eventually require kidney transplantation. The selection of RRT should be made by evaluating the entire clinical scenario, the presence of factors that can be modified with RRT, and trends of laboratory tests when deciding to start RRT instead of relying on blood urea nitrogen (BUN) and creatinine thresholds alone [1–3]. In addition to guidelines, individualized factors, including the patient’s financial situation, compliance, caregivers, and residual urine output, should also be considered.

Still, whether nephrologists are well acquainted with the principles of RRT and apply them in practice is poorly understood. Emotional burden is often encountered during and influences RRT decision-making [4, 5]. It has been reported that many nephrologists feel uncomfortable not offering dialysis for reasons they poorly understand, but it might include prognostic uncertainty and discomfort with a possible death [5, 6]. The Knowledge, Attitude, and Practice (KAP) framework is a quantitative method based on standardized questionnaires that provide quantitative and qualitative data that can help unravel the misconceptions and misunderstandings posing obstacles in a specific clinical activity and help identify specific points for behavior changes [7, 8].

Therefore, this study aimed to examine the KAP of nephrologists on the decision of RRT. The results could help pinpoints areas that would benefit from additional training.

## Methods

### Study design and participants

This multicenter cross-sectional study was conducted between July 1, 2022, and August 20, 2022, and enrolled all qualified nephrologists who volunteered to participate. Nephrologists on maternity or sick leave or temporary workers were excluded. This study was approved by the Ethics Committee of the Hunan Provincial People’s Hospital (as the lead center). All participants signed the informed consent.

### Procedures

According to the 17^th^ Acute Disease Quality Initiative International Consensus Conference: introducing precision renal replacement therapy and KDIGO clinical practice guideline for acute kidney injury [9]. This questionnaire was modified following the comments of two senior nephrologists. Initially, the knowledge dimension consisted of 12 questions, while the attitudes and practices dimensions had 12 and 10 questions, respectively. After consultation with experts, the knowledge dimension was reviewed, and four questions were added, resulting in 16 questions. The attitude dimension was expanded with seven more questions, resulting in 19. The practice dimension was modified to assess all three decision-making settings, and some of the questions were deemed inappropriate or inaccurate and were replaced. For instance, the original questionnaire referred to “palliative care specialists,” which may not be available in some areas. Hence, it was modified to “nutrition specialists,” resulting in 11 questions.

The questionnaire was pre-tested and had a Cronbach’s α of 0.938 and a KMO of 0.885, suggesting high internal consistency. The final questionnaire was the Chinese version and included four dimensions: 1) the demographic data of the participants, including gender, age, marital status, level of education, grade of hospitals, professional title, working experience, methods of RRT available in hospitals, population with dialysis (including peritoneal dialysis and hemodialysis) in hospitals, and region of hospitals; 2) 16 questions about the knowledge for the decision of RRT (scored 1 point for correct answers and 0 points for incorrect or unclear answers, ranging from 0 to 16 points); 3) 19 items (5-point Likert scale) about the attitude of nephrologist on the decision of RRT, with 15 questions scored from very positive (5 points) to very negative (1 point) (total score ranging from 15 to 75 points) and four open questions; 4) 11 items on the practice of nephrologist on decision of RRT, including six questions scored on a 5-point Likert scale scored from always (5 points) to never (1 point) (total score ranging from 6 to 30 points) and five open questions.

An online questionnaire with a QR code was established using the WeChat-based Questionnaire Star applet to collect data through WeChat. The participants logged in via WeChat by scanning the QR code and completing the questionnaire. In order to ensure quality and completeness, a given IP address could only submit the questionnaire once, and all questions had to be answered. The research team checked all questionnaires for completeness, internal coherence, and rationality.

### Statistical analysis

SPSS 26.0 (IBM Corp., Armonk, NY, USA) was used for statistical analysis. Continuous data were expressed as means ± standard deviation (SD) and compared by t-test or ANOVA, while the least-significant difference (LSD) was used as a post hoc test. Categorical data were expressed as n (%). Pearson correlation was used to analyze the correlations between knowledge, attitude, and practice scores. Logistic regression was used to analyze influencing factors of practice. The median of the practice score (= 20) was used as the cut-off value. Factors with *P* < 0.001 in the univariable analyses were included in the multivariable logistic regression analysis. Two-sided *P*-values < 0.05 were considered statistically significant.

## Results

Ultimately, 327 nephrologists participated in this study, including 104 (31.80%) males and 223 (68.20%) females. Most were 31–40 years of age (61.16%), unmarried (86.54%), bachelor’s degree education (57.49%), public tertiary hospital (65.44%), with intermediate professional titles (51.68%), and with 5–10 years of working experience (35.17%). Peritoneal dialysis was available for 78.59% of the nephrologists, hemodialysis for 98.47%, kidney transplantation for 23.55%, and none for 1.53%. Most nephrologists had a patient volume of 100–300 per year (37.31%) (Table 1).

**Table 1:** Baseline characteristics and KAP scores

Variables	N (%)	Knowledge scores		Attitude scores		Practice scores	
		Mean ± SD	P	Mean ± SD	P	Mean ± SD	P
**Total scores**	327 (100.00%)	12.03 ± 2.11		58.39 ± 6.62		27.15 ± 2.74	
**Gender**			0.086		0.201		0.759
Male	104 (31.80%)	12.33 ± 1.79		57.70 ± 6.14		27.22 ± 2.69	
Female	223 (68.20%)	11.90 ± 2.23		58.71 ± 6.82		27.12 ± 2.77	
**Age (years)**			0.198		0.293		0.314
< 30	52 (15.90%)	11.69 ± 2.91		59.46 ± 8.29		27.19 ± 2.69	
31–40	200 (61.16%)	11.97 ± 1.98		58.50 ± 6.47		27.05 ± 2.68	
41–50	58 (17.74%)	12.36 ± 1.87		57.07 ± 5.59		27.12 ± 3.07	
> 50	17 (5.20%)	12.71 ± 1.10		58.35 ± 5.48		28.35 ± 2.29	
**Marital status**			0.417		0.627		0.452
Unmarried	283 (86.54%)	12.08 ± 1.99		58.32 ± 6.45		27.20 ± 2.69	
Married (married + divorced + widowed)	44 (13.46%)	11.73 ± 2.76		58.84 ± 7.68		26.86 ± 3.04	
**Level of education**			< 0.001		0.063		0.578
Bachelor’s degree	188 (57.49%)	11.61 ± 2.48		59.12 ± 7.20		27.03 ± 2.81	
Master’s degree	121 (37.00%)^a^	12.57 ± 1.22		57.32 ± 5.88		27.27 ± 2.70	
Doctorate	18 (5.50%)	12.83 ± 1.54		57.94 ± 3.39		27.61 ± 2.23	
**Grade of hospitals**			< 0.001		0.987		0.072
Below public tertiary hospital	87 (26.61%)	11.51 ± 2.45		58.43 ± 7.66		27.01 ± 2.79	
Public tertiary hospital	214 (65.44%)^b^	12.39 ± 1.68		58.40 ± 5.93		27.34 ± 2.67	
Private hospital	26 (7.95%)^c^	10.88 ± 3.15		58.19 ± 8.31		26.08 ± 2.91	
**Professional title**			< 0.001		0.059		0.124
Junior	71 (21.71%)	11.45 ± 2.71		59.65 ± 7.74		27.30 ± 2.91	
Intermediate	169 (51.68%)	11.90 ± 2.03		58.52 ± 6.41		26.98 ± 2.63	
Associate senior	68 (20.80%)^d, e^	12.65 ± 1.50		56.65 ± 5.97		27.06 ± 2.95	
Senior	19 (5.81%)^f, g^	13.21 ± 0.92		58.74 ± 5.02		28.53 ± 1.90	
**Working experience**			0.258		0.224		0.646
≤ 5 years	62 (18.96%)	12.15 ± 2.13		59.15 ± 6.36		26.87 ± 2.83	
5–10 years	115 (35.17%)	12.03 ± 1.96		57.58 ± 6.42		27.30 ± 2.49	
11–15 years	69 (21.10%)	11.62 ± 2.56		59.42 ± 7.47		26.96 ± 2.82	
≥ 16 years	81 (24.77%)	12.30 ± 1.85		58.07 ± 6.24		27.32 ± 2.96	
**Methods of renal replacement therapy available in hospitals**							
Peritoneal dialysis	257 (78.59%)	12.28 ± 1.80	< 0.001	58.33 ± 6.11	0.763	27.34 ± 2.66	0.018
Hemodialysis	322 (98.47%)	12.11 ± 1.92	< 0.001	58.34 ± 6.55	0.306	27.20 ± 2.69	0.006
Kidney transplantation	77 (23.55%)	12.74 ± 1.30	< 0.001	57.48 ± 5.49	0.169	27.73 ± 2.47	0.035
None	5 (1.53%)	7.00 ± 5.92	< 0.001	61.40 ± 10.71	0.306	23.80 ± 4.09	0.006
**Population with dialysis (including peritoneal dialysis and hemodialysis) in hospitals**			< 0.001		0.719		0.058
< 100	32 (9.79%)	10.53 ± 3.51		59.56 ± 7.85		26.22 ± 3.24	
100–300	122 (37.31%)^h^	11.87 ± 2.15		58.48 ± 7.10		26.91 ± 2.72	
300–500	82 (25.08%)^i^	12.07 ± 1.65		58.10 ± 5.22		27.45 ± 2.92	
> 500	91 (27.83%)^j, k^	12.75 ± 1.38		58.11 ± 6.67		27.54 ± 2.31	
**Region of hospitals**			0.556		0.589		0.730
Northeastern region	1 (0.31%)	10.00		58.00		25.00	
Eastern Region	19 (5.81%)	12.16 ± 1.86		57.42 ± 6.16		27.63 ± 2.59	
Central Region	300 (91.74%)	12.01 ± 2.15		58.52 ± 6.67		27.12 ± 2.76	
Western Region	7 (2.14%)	12.86 ± 0.69		55.43 ± 5.80		27.43 ± 2.57	

^a^Bachelor’s degree vs. Master’s degree, *P* < 0.001

^b^Below public tertiary hospital vs. Public tertiary hospital, *P* = 0.001

^c^Public tertiary hospital vs. Private hospital, *P* < 0.001

^d^Intermediate vs. Associate senior, *P* = 0.001

^e^Public tertiary hospital vs. Private hospital, *P* < 0.001

^f^Below public tertiary hospital vs. Public tertiary hospital, *P* = 0.001

^g^Public tertiary hospital vs. Private hospital, *P* < 0.001

^h^ < 100 vs. 100–300, *P* = 0.001

^i^ < 100 vs. 300–500, *P* < 0.001

^j^ < 100 vs. > 500, *P* < 0.001

^k^100-300 vs. > 500, *P* = 0.002 [one-way ANOVA with Fisher’s Least Significant Difference (LSD) Test]

The total knowledge, attitude, and practice scores were 12.03 ± 2.11/16, 58.39 ± 6.62/75, and 27.15 ± 2.74/30, respectively. The knowledge scores were influenced by the level of education (*P* < 0.001), grade of hospitals (*P* < 0.001), professional title (*P* < 0.001), the available RRT methods (all *P* < 0.001), and patient volume (*P* < 0.001). No factor was significantly associated with the attitude scores (all *P* > 0.05). The available RRT methods were associated with the practice scores (all *P* < 0.05) (Table 1). Table 2 presents the responses to the knowledge dimension items. Table 3 presents the answers to the items of the attitude dimension, while Table 4 presents the answers to the practice dimension items. The Pearson correlation analyses showed that the knowledge scores correlated with the practice scores (*r* = 0.239, *P* < 0.001) and the consideration score of peritoneal dialysis (*r* = 0.171, *P* = 0.002). The attitude scores correlated with the practice scores (*r* = 0.251, *P* < 0.001), the consideration score of peritoneal dialysis (*r* = 0.453, *P* < 0.001), the consideration score of hemodialysis (*r* = 0.425, *P* < 0.001), and the consideration score of kidney transplantation (*r* = 0.407, *P* < 0.001). The practice scores correlated with the consideration score of peritoneal dialysis (*r* = 0.145, *P* = 0.009) and the consideration score of kidney transplantation (*r* = 0.119, *P* = 0.031). The consideration score of peritoneal dialysis correlated with the consideration score of hemodialysis (*r* = 0.597, *P* < 0.001) and the consideration score of kidney transplantation (*r* = 0.489, *P* < 0.001). The consideration score of hemodialysis correlated with the consideration score of kidney transplantation (*r* = 0.673, *P* < 0.001) (Supplementary Table 1).

**Table 2:** “Knowledge” dimension

Knowledge	N (%)		
	**True**	**False**	**Unclear**
1. Renal replacement therapy (RRT) includes peritoneal dialysis, hemodialysis, plasma exchange, hemoperfusion, continuous renal replacement therapy, multiple heterozygous modalities, and kidney transplantation	318 (97.25%)	7 (2.14%)	2 (0.61%)
2. The initiation of RRT should be individualized rather than relying solely on renal function indicators or AKI grading	313 (95.72%)	4 (1.22%)	10 (3.06%)
3. Removing RRT depends solely on the recovery of kidney function	237 (72.48%)	71 (21.71%)	19 (5.81%)
4. To understand ongoing renal recovery, it is recommended to monitor urine output and creatinine during RRT. Besides, urine output is more valuable than creatinine	244 (74.62%)	61 (18.65%)	22 (6.73%)
5. Internal jugular and sub-femoral veins are preferred for intubation in RRT, and ultrasound-guided catheter placement is recommended	290 (88.69%)	26 (7.95%)	11 (3.36%)
6. The initiation and frequency of dialysis for patients with uremia should make full use of available technology, which should be balanced from economics, patient survival, and quality of life	319 (97.55%)	4 (1.22%)	4 (1.22%)
7. The initiation of dialysis should be determined by whether the patient has symptoms of uremia or internal environmental disturbances that do not respond to medical therapy rather than relying solely on the glomerular filtration rate	313 (95.72%)	7 (2.14%)	7 (2.14%)
8. Premature dialysis is detrimental to the protection of residual kidney function, may increase the risk of dialysis-related complications and mortality, and cause a waste of medical resources	270 (82.57%)	49 (14.98%)	8 (2.45%)
9. Late dialysis can lead to increased complications, including longer hospital stays, increased hospitalizations, increased risk of death, and increased medical costs	319 (97.55%)	5 (1.53%)	3 (0.92%)
10. Dialysis is started when patients with eGFR < 15 mL/min/1.73m^2^ and uncontrollable symptoms of uremia	284 (86.85%)	29 (8.87%)	14 (4.28%)
11. Patients with high risk (e.g., with diabetes) do not require early initiation of dialysis therapy	43 (13.15%)	271 (82.87%)	13 (3.98%)
12. Regardless of clinical symptoms, patients with eGFRs < 6 mL/min/1.73 m^2^ should be started on dialysis	169 (51.68%)	135 (41.28%)	23 (7.03%)
13. Actual GFR is overestimated under using creatinine clearance to indicate the renal function of older adults with end-stage renal disease (ESRD)	249 (76.15%)	46 (14.07%)	32 (9.79%)
14. For critically ill patients with AKI stage 3, RRT is indicated by increased blood urea nitrogen over 140 mg/dl or complications	47 (14.37%)	247 (75.54%)	33 (10.09%)
15. For patients who choose dialysis therapy, the decision to start maintenance dialysis can be based on specific levels of renal function alone	255 (77.98%)	62 (18.96%)	10 (3.06%)
16. Patients with dialysis therapy should gradually increase the dialysis dose and do not advocate with high-frequency dialysis at the beginning	265 (81.04%)	53 (16.21%)	9 (2.75%)

**Table 3:** “Attitude” dimension

To what extent do the following factors of a patient’s in clinical practice influence your decision to proceed with renal replacement therapy?	Extremely	Comparatively	Generally	Comparatively not	Completely not
Age	32 (9.79%)	157 (48.01%)	105 (32.11%)	29 (8.87%)	4 (1.22%)
Gender	10 (3.06%)	31 (9.48%)	53 (16.21%)	101 (30.89%)	132 (40.37%)
Laboratory indicators	123 (37.61%)	167 (51.07%)	34 (10.40%)	1 (0.31%)	2 (0.61%)
Nutritional status	66 (20.18%)	200 (61.16%)	57 (17.43%)	4 (1.22%)	
Clinical symptoms	197 (60.24%)	118 (36.09%)	10 (3.06%)	1 (0.31%)	1 (0.31%)
Pre-existing illnesses	87 (26.61%)	191 (58.41%)	42 (12.84%)	7 (2.14%)	
Comorbidities	181 (55.35%)	130 (39.76%)	15 (4.59%)	1 (0.31%)	
Patient’s understanding of renal replacement therapy	41 (12.54%)	154 (47.09%)	113 (34.56%)	17 (5.20%)	2 (0.61%)
Patient’s personal willingness	77 (23.55%)	184 (56.27%)	60 (18.35%)	6 (1.83%)	
Adherence	73 (22.32%)	207 (63.30%)	42 (12.84%)	5 (1.53%)	
Family caregiving	53 (16.21%)	212 (64.83%)	54 (16.51%)	8 (2.45%)	
Economic condition	59 (18.04%)	192 (58.72%)	63 (19.27%)	13 (3.98%)	
Psychological and mental status	60 (18.35%)	208 (63.61%)	56 (17.13%)	3 (0.92%)	
Quality of life	53 (16.21%)	219 (66.97%)	49 (14.98%)	6 (1.83%)	
Experience of dialysis center	50 (15.29%)	192 (58.72%)	66 (20.18%)	14 (4.28%)	5 (1.53%)
**What is your attitude towards the following kidney replacement therapies?**	**Extremely positive**	**Relatively positive**	**Generally**	**Relatively negative**	**Extremely negative**
Hemodialysis	82 (25.08%)	203 (62.08%)	40 (12.23%)	2 (0.61%)	
Peritoneal dialysis	66 (20.18%)	175 (53.52%)	81 (24.77%)	5 (1.53%)	
Kidney transplantation	54 (16.51%)	125 (38.23%)	138 (42.20%)	10 (3.06%)	
Conservative Treatment	36 (11.01%)	94 (28.75%)	161 (49.24%)	33 (10.09%)	3 (0.92%)

**Table 4:** “Practice” dimension

Statement	Extremely compatible	Comparatively compatible	Generally compatible	Comparatively incompatible	Extremely incompatible
1. When making decisions, I will try to move away from a “one-size-fits-all” approach to dialysis and provide a more individualized approach to care that takes into account the patient’s goals and preferences while maintaining quality and safety	201 (61.47%)	110 (33.64%)	14 (4.28%)	1 (0.31%)	1 (0.31%)
2. I will recommend that patients and their families receive dialysis-related education	256 (78.29%)	66 (20.18%)	5 (1.53%)		
3. I will discuss the risks and overall prognosis of end-stage renal disease with the patient and provide appropriate treatment options with reference to the patient’s choices	230 (70.34%)	87 (26.61%)	9 (2.75%)	1 (0.31%)	
4. If the patient decides to “postpone” the dialysis decision, or is temporarily unable to make a decision, I will make it clear that a similar decision can be made again in the future	193 (59.02%)	114 (34.86%)	15 (4.59%)	4 (1.22%)	1 (0.31%)
5. When a patient chooses not to receive dialysis treatment, in addition to CKD management, I will contact a nutritional specialist to provide advice on slowing disease progression	147 (44.95%)	113 (34.56%)	52 (15.90%)	13 (3.98%)	2 (0.61%)
6. When a patient has a significant decrease in eGFR (e.g., < 10 ml/min/1.73 m^2^), early symptoms of uremia, or other significant changes (e.g., acute kidney injury, hospitalization, etc.), palliative dialysis treatment, time-limited dialysis treatment, permanent dialysis treatment, and palliative treatment without dialysis should be considered. I will recommend permanent dialysis treatment, but will respect the patient’s other choices	171 (52.29%)	140 (42.81%)	11 (3.36%)	4 (1.22%)	1 (0.31%)
7. In what ways does your department make patients more aware of information about renal replacement therapy? (Multiple choice)					
Departmental education	317 (96.94%)				
Science activities (online classes, etc.)	250 (76.45%)				
WeChat official account	218 (66.67%)				
Others	58 (17.74%)				
**To what extent do the following factors influence your decision making about peritoneal dialysis?**	**Extremely**	**Comparatively**	**Generally**	**Comparatively not**	**Completely not**
Underlying Diseases	146 (44.65%)	161 (49.24%)	19 (5.81%)	1 (0.31%)	
Comorbidities	150 (45.87%)	160 (48.93%)	17 (5.20%)		
Residual urine volume	156 (47.71%)	152 (46.48%)	19 (5.81%)		
Age	87 (26.61%)	171 (52.29%)	64 (19.57%)	5 (1.53%)	
Abdominal condition	170 (51.99%)	138 (42.20%)	18 (5.50%)	1 (0.31%)	
Economic situation	66 (20.18%)	181 (55.35%)	76 (23.24%)	4 (1.22%)	
Transportation convenience	86 (26.30%)	177 (54.13%)	59 (18.04%)	3 (0.92%)	2 (0.61%)
The level of patient knowledge	106 (32.42%)	180 (55.05%)	39 (11.93%)	2 (0.61%)	
Home hygiene conditions	157 (48.01%)	153 (46.79%)	15 (4.59%)	2 (0.61%)	
**To what extent do the following factors influence your decision making about Hemodialysis?**	**Extremely**	**Comparatively**	**Generally**	**Comparatively not**	**Completely not**
Underlying Diseases	139 (42.51%)	166 (50.76%)	20 (6.12%)	1 (0.31%)	1 (0.31%)
Comorbidities	157 (48.01%)	158 (48.32%)	11 (3.36%)		1 (0.31%)
Residual urine volume	95 (29.05%)	164 (50.15%)	54 (16.51%)	13 (3.98%)	1 (0.31%)
Age	75 (22.94%)	166 (50.76%)	73 (22.32%)	9 (2.75%)	4 (1.22%)
Peripheral vascular conditions	173 (52.91%)	145 (44.34%)	9 (2.75%)		
Economic situation	65 (19.88%)	186 (56.88%)	72 (22.02%)	3 (0.92%)	1 (0.31%)
Transportation convenience	89 (27.22%)	174 (53.21%)	61 (18.65%)	2 (0.61%)	1 (0.31%)
The level of patient knowledge	63 (19.27%)	150 (45.87%)	96 (29.36%)	14 (4.28%)	4 (1.22%)
Home hygiene conditions	60 (18.35%)	127 (38.84%)	103 (31.50%)	31 (9.48%)	6 (1.83%)
**To what extent do the following factors influence your decision making about Kidney transplantation?**	**Extremely**	**Comparatively**	**Generally**	**Comparatively not**	**Completely not**
Underlying Diseases	164 (50.15%)	147 (44.95%)	16 (4.89%)		
Comorbidities	166 (50.76%)	143 (43.73%)	18 (5.50%)		
Renal source	163 (49.85%)	140 (42.81%)	23 (7.03%)	1 (0.31%)	
Age	184 (56.27%)	134 (40.98%)	9 (2.75%)		
Economic situation	55 (16.82%)	122 (37.31%)	97 (29.66%)	42 (12.84%)	11 (3.36%)
Transportation convenience	83 (25.38%)	161 (49.24%)	63 (19.27%)	17 (5.20%)	3 (0.92%)
The level of patient knowledge	68 (20.80%)	141 (43.12%)	93 (28.44%)	22 (6.73%)	3 (0.92%)
Home hygiene conditions	204 (62.39%)	114 (34.86%)	8 (2.45%)	1 (0.31%)	

The multivariable logistic regression analysis showed that the attitude score (OR = 1.19, 95%CI: 1.13–1.25, *P* < 0.001), 41–50 years of age (vs. < 30, OR = 0.45, 95%CI: 0.21–0.98, *P* = 0.045), and > 50 years of age (vs. < 30, OR = 0.27, 95%CI: 0.08–0.84, *P* = 0.024) were independently associated with the consideration score of peritoneal dialysis (Table 5).

**Table 5:** Logistic regression (consideration scores of peritoneal dialysis, hemodialysis, and kidney transplantation)

Factors	Consideration score of peritoneal dialysis		Consideration score of hemodialysis		Consideration score of kidney transplantation	
	OR (95%CI)	P	OR (95%CI)	P	OR (95%CI)	P
**Knowledge score**	1.08 (0.97, 1.20)	0.161	0.98 (0.89, 1.09)	0.751	0.89 (0.79, 1.00)	0.041
**Attitude score**	1.19 (1.13, 1.25)	< 0.001	1.14 (1.09, 1.19)	< 0.001	1.12 (1.07, 1.16)	< 0.001
**Gender**						
Male	Reference		Reference		Reference	
Female	1.34 (0.84, 2.15)	0.217	1.66 (1.04, 2.66)	0.034	1.37 (0.86, 2.19)	0.190
**Age (year)**						
< 30	Reference		Reference		Reference	
31–40	0.69 (0.36, 1.30)	0.249	0.59 (0.31, 1.13)	0.112	0.61 (0.32, 1.18)	0.143
41–50	0.45 (0.21, 0.98)	0.045	0.27 (0.12, 0.60)	0.001	0.45 (0.20, 0.97)	0.042
> 50	0.27 (0.08, 0.84)	0.024	0.31 (0.10, 0.97)	0.043	0.24 (0.08, 0.77)	0.016
**Marital status**						
Unmarried	Reference		Reference		Reference	
Married(Married + divorced + widowed)	0.72 (0.37, 1.39)	0.332	0.73 (0.38, 1.40)	0.344	0.91 (0.48, 1.74)	0.784
**Level of education**						
Bachelor	Reference		Reference		Reference	
Master	0.80 (0.50, 1.26)	0.331	0.87 (0.55, 1.37)	0.547	0.75 (0.48, 1.20)	0.230
Doctor	1.42 (0.51, 3.94)	0.503	0.77 (0.29, 2.04)	0.603	0.52 (0.20, 1.38)	0.188
**Grade of hospitals**						
Below public tertiary hospital	0.98 (0.59, 1.62)	0.940	1.18 (0.71, 1.95)	0.532	2.22 (0.34, 1.73)	0.004
Public tertiary hospital	Reference		Reference		Reference	
Private hospital	0.62 (0.28, 1.41)	0.255	0.61 (0.27, 1.39)	0.236	0.77 (0.34, 1.73)	0.522
**Professional title**						
Junior	Reference		Reference		Reference	
Intermediate	1.00 (0.57, 1.76)	0.998	0.74 (0.42, 1.31)	0.305	0.75 (0.42, 1.33)	0.327
Associate senior	0.73 (0.37, 1.43)	0.363	0.51 (0.26, 1.01)	0.054	0.54 (0.28, 1.07)	0.079
Senior	0.62 (0.23, 1.72)	0.359	0.34 (0.12, 0.96)	0.042	0.49 (0.18, 1.36)	0.171
**Working experience**						
≤ 5 years	Reference		Reference		Reference	
5–10 years	0.88 (0.47, 1.66)	0.696	0.77 (0.41, 1.45)	0.411	0.85 (0.45, 1.60)	0.615
11–15 years	1.05 (0.52, 2.12)	0.904	0.81 (0.40, 1.64)	0.565	1.18 (0.58, 2.41)	0.642
≥ 16 years	0.56 (0.29, 1.09)	0.089	0.45 (0.23, 0.88)	0.020	0.56 (0.29, 1.09)	0.089
**Methods of renal replacement therapy available in hospitals**						
Peritoneal dialysis	1.53 (0.90, 2.61)	0.115	0.95 (0.56, 1.62)	0.854	0.69 (0.40, 1.19)	0.177
Hemodialysis	2.00 (0.33, 12.13)	0.451	0.80 (0.13, 4.87)	0.812	0.33 (0.04, 2.98)	0.323
Kidney transplantation	0.95 (0.57, 1.58)	0.833	0.75 (0.45, 1.26)	0.278	0.66 (0.40, 1.10)	0.113
None	0.50 (0.08, 3.03)	0.451	1.24 (0.21, 7.55)	0.812	3.04 (0.34, 27.49)	0.323
**Population with dialysis (including peritoneal dialysis and hemodialysis) in hospitals**						
< 100	Reference		Reference		Reference	
100–300	0.89 (0.40, 1.97)	0.774	0.64 (0.28, 1.44)	0.278	0.61 (0.27, 1.40)	0.246
300–500	0.87 (0.38, 2.00)	0.751	0.52 (0.22, 1.22)	0.135	0.58 (0.24, 1.38)	0.219
> 500	0.91 (0.40, 2.07)	0.826	0.64 (0.28, 1.48)	0.295	0.53 (0.23, 1.25)	0.145

The median of the consideration scores of peritoneal dialysis, hemodialysis, and kidney transplantation was used as the cut-off value

The attitude score (OR = 1.14, 95%CI: 1.09–1.19, *P* < 0.001), female gender (OR = 1.66, 95%CI: 1.04–2.66, *P* = 0.034), 41–50 years of age (vs. < 30, OR = 0.27, 95%CI: 0.12–0.60, *P* = 0.001), > 50 years of age (vs. < 30, OR = 0.31, 95%CI: 0.10–0.97, *P* = 0.043), senior professional title (vs. junior, OR = 0.34, 95%CI: 0.12–0.96, *P* = 0.042), and ≥ 16 years of working experience (vs. ≤ 5 years, OR = 0.45, 95%CI: 0.23–0.88, *P* = 0.020) were independently associated with the consideration score of hemodialysis (Table 5).

The knowledge score (OR = 1.89, 95%CI: 0.79–0.995, *P* = 0.041), attitude score (OR = 1.12, 95%CI: 1.07–1.16, *P* < 0.001), 41–50 years of age (vs. < 30, OR = 0.45, 95%CI: 0.20–0.97, *P* = 0.042), > 50 years of age (vs. < 30, OR = 0.24, 95%CI: 0.08–0.77, *P* = 0.016), and below public tertiary hospital (vs. public tertiary hospital, OR = 2.22, 95%CI: 1.30–3.79, *P* = 0.004) were independently associated with the consideration score of kidney transplantation (Table 5).

The multivariable logistic regression analysis showed that the knowledge scores (OR = 1.08, 95%CI: 1.03–1.13, *P* = 0.001) were independently associated with the practice scores (Table 6).

**Table 6:** Logistic regression (practice)

Factors	Univariable logistic regression		Multivariable logistic regression	
	OR (95%CI)	P	OR (95%CI)	P
Knowledge score	1.12 (1.00, 1.25)	0.057	1.08 (1.03, 1.13)	0.001
Attitude score	1.07 (1.04, 1.11)	< 0.001	1.12 (0.99, 1.27)	0.077
Consideration score of peritoneal dialysis	1.15 (1.00, 1.31)	0.043	0.96 (0.80, 1.15)	0.634
Consideration score of hemodialysis	1.09 (1.00, 1.19)	0.057	1.00 (0.87, 1.14)	0.972
Consideration score of kidney transplantation	1.15 (1.02, 1.29)	0.022	1.08 (0.92, 1.28)	0.347
**Gender**				
Male	Reference			
Female	0.84 (0.52, 1.34)	0.455		
**Age (year)**				
< 30	Reference			
31–40	0.79 (0.43, 1.46)	0.452		
41–50	1.10 (0.52, 2.33)	0.807		
> 50	2.31 (0.74, 7.19)	0.148		
**Marital status**				
Unmarried	Reference			
Married(Married + divorced + widowed)	1.06 (0.56, 2.03)	0.852		
**Level of education**				
Bachelor	Reference			
Master	1.11 (0.70, 1.76)	0.652		
Doctor	0.53 (0.18, 1.55)	0.247		
**Grade of hospitals**				
Below public tertiary hospital	0.79 (0.47, 1.31)	0.359		
Public tertiary hospital	Reference			
Private hospital	0.55 (0.23, 1.31)	0.176		
**Professional title**				
Junior	Reference			
Intermediate-grade	0.66 (0.38, 1.16)	0.151		
Associate senior	0.72 (0.37, 1.41)	0.332		
Senior	2.36 (0.81, 6.90)	0.117		
**Working experience**				
≤ 5 years	Reference			
5–10 years	1.30 (0.69, 2.46)	0.411		
11–15 years	1.03 (0.51, 2.08)	0.945		
≥ 16 years	1.58 (0.80, 3.09)	0.187		
**Methods of renal replacement therapy available in hospitals**				
Peritoneal dialysis	1.66 (0.95, 2.89)	0.076	1.17 (0.57, 2.39)	0.668
Hemodialysis	2.96 (0.33, 26.80)	0.334		
Kidney transplantation	1.37 (0.82, 2.28)	0.235		
None	0.34 (0.04, 3.05)	0.334		
**Population with dialysis (including peritoneal dialysis and hemodialysis) in hospitals**				
< 100	Reference		Reference	
100–300	1.24 (0.54, 2.86)	0.612	1.14 (0.443, 2.92)	0.789
300–500	2.43 (1.02, 5.76)	0.044	2.28 (0.80, 6.52)	0.125
> 500	1.80 (0.77, 4.24)	0.176	1.55 (0.54, 4.46)	0.419
**Region of hospitals**				
Northeastern region	*	*		
Eastern Region	1.56 (0.61, 3.94)	0.351		
Central Region	Reference			
Western Region	1.05 (0.23, 4.77)	0.950		

^*^due to small sample size

## Discussion

This study suggests that good attitudes may lead nephrologists to make more considerations when choosing between peritoneal dialysis, hemodialysis, and kidney transplantation, while senior physicians may consider relatively less when making decisions, and in addition, both good knowledge and good attitudes may lead to good practice.

In the present study, the knowledge, attitude, and practice scores of Chinese nephrologists regarding the decision for RRT were 12.0 ± 2.1/16, 58.4 ± 6.6/75, and 27.2 ± 2.7/30, respectively, which could be considered moderate, low, and high. Ockhuis & Kyriacos [10] (South Africa) reported knowledge, attitude, and practice scores of 10.8 ± 3.1/18, 25.9 ± 6.0/41, and 35.8 ± 6.0/50 for the safe use of unfractionated heparin during RRT. A study in Nepal showed relatively poor to moderate knowledge regarding kidney diseases but relatively good attitude and practice [11]. A study in Pakistan revealed that only 18% of physicians had good knowledge about kidney diseases [12], while good knowledge was seen in 24% of physicians in Sudan [13]. The need for training of Brazilian palliative care physicians regarding RRT has been emphasized by a study [14]. The relatively higher KAP scores observed in the presented study could be because only nephrologists were enrolled.

In this study, only the knowledge scores independently influenced the practice scores. On the other hand, since peritoneal dialysis, hemodialysis, and kidney transplantation have different indications and target patient populations, more factors were associated with those three scores: the attitude score and age were independently associated with the consideration score of peritoneal dialysis; the attitude score, female gender, age, senior professional title, and ≥ 16 years of working experience were independently associated with the consideration score of hemodialysis; the knowledge score, attitude score, age, and below public tertiary hospital were independently associated with the consideration score of kidney transplantation. Ochkuis & Kyriacos [10] reported that the category of professionals, knowledge, and years of experience influenced the quality of dialysis practice, but their study focused on using unfractionated heparin. The middle-adulthood age range (40–65 years) is considered stage 7 of psychosocial development, characterized by an experience of stagnation and a feeling of unproductiveness [15, 16], which could explain why older age is consistently associated with lower KAP scores. A qualitative study by Greer et al. identified hospital resources, provider skills, and patient attitudes as the main barriers to RRT [17]. Wu et al. [18] reported that the main barriers to implementing AKI management in China were inadequate knowledge, inadequate training, absence of clinical protocols, and insufficient multidisciplinary cooperation. It is also supported by studies from developing countries [19–21]. A worldwide survey identified the patients, the nephrologists, geography, and the healthcare systems as barriers to RRT in 78%, 71%, 72%, and 73% of the countries, respectively [22]. Work experience was not associated with the KAP dimensions, which was a little surprising. Indeed, it can be expected that one’s knowledge will increase with experience, but work experience is only a metric of how long an individual has been involved in a particular work. It does not evaluate his knowledge, motivation, enthusiasm, and attitude. A young physician can be very enthusiastic at the idea of helping patients, while another might be only attracted by the social status of being a physician. Some older physicians can still be very enthusiastic in their job, while others might be tired and waiting for retirement. Another factor could be that continuous education, favorable attitudes, and practice according to the guidelines are similar across work experience. Unfortunately, the available data do not allow delving deeper into why work experience is not associated with any KAP dimensions.

Hence, the present study identified categories of nephrologists who might benefit from training on RRT to improve their KAP. Considering that the current clinical trend is that the selection of RRT should be made by evaluating the entire clinical scenario, modifiable factors, and laboratory tests when deciding to start RRT instead of relying on BUN and creatinine alone [1–3], training should be implemented to improve the nephrologists’ awareness of the factors to be considered for starting RRT. Group discussions to share past experiences could be of use. Postgraduate training and continuous education should be emphasized. Multidisciplinary management should also be explored.

This study has some limitations. Considering the number of nephrologists in China, the sample size was relatively small, and the nephrologists were mainly from Eastern China. Future studies should enroll a sample size more representative of the entire country. The most effective treatment for CKD remains kidney transplantation, but only a few questions were about transplantation. Of course, a KAP survey has limitations. It can only identify deficits related to the asked questions, and the points not covered by the questionnaire items will remain unknown. In addition, a KAP questionnaire is usually specifically designed for a given hospital, province, or country, and the results are difficult to generalize. Nevertheless, they can give ideas to researchers from around the globe for performing KAP surveys and implementing improvement training.

In conclusion, the knowledge, attitude, and practice scores of nephrologists regarding RRT were moderate, low, and high, respectively. This study also revealed factors associated with the KAP of nephrologists regarding RRT. It identified areas that could be targeted by additional training. Future studies should examine the implementation of different training methods to improve the KAP of nephrologists toward RRT.

## Supplementary Information

**Additional file 1.**

## Data Availability

All data generated or analyzed during this study are included in this published article [and its supplementary information files].

## Abbreviations

KAP: Knowledge, Attitude, and Practice
RRT: Renal replacement therapy
CRRT: Continuous renal replacement therapy
IRRT: Intermittent renal replacement therapy
SLED: Sustained low-efficiency dialysis
BUN: Blood urea nitrogen
SD: Standard deviation
