# Supplementary material for: Knowledge, attitude, and practice of nephrologists on the decision for renal replacement therapy
Source: BMC Public Health. 2023 Apr 5;23:654. doi: 10.1186/s12889-023-15530-0 (PMC10077733; doi:10.1186/s12889-023-15530-0)
Supplement: Supplementary file 1 — Additional file 1. [file 12889_2023_15530_MOESM1_ESM.docx]

**Supplementary Table 1.** Pearson correlation analysis.

|  | Knowledge | Attitude | Practice | Consideration score of peritoneal dialysis | Consideration score of hemodialysis | Consideration score of kidney transplantation |
| --- | --- | --- | --- | --- | --- | --- |
| Knowledge | 1 |  |  |  |  |  |
| Attitude | -0.03 (P=0.606) | 1 |  |  |  |  |
| Practice | 0.24 (P<0.001) | 0.25 (P<0.001) | 1 |  |  |  |
| Consideration score of peritoneal dialysis | 0.17 (P=0.002) | 0.45 (P<0.001) | 0.15 (P=0.009) | 1 |  |  |
| Consideration score of hemodialysis | 0.01 (P=0.867) | 0.43 (P<0.001) | 0.11 (P=0.052) | 0.60 (P<0.001) | 1 |  |
| Consideration score of kidney transplantation | -0.06 (P=0.277) | 0.41 (P<0.001) | 0.12 (P=0.031) | 0.49 (P<0.001) | 0.67 (P<0.001) | 1 |
